# Application of close‐kin mark–recapture to an American black bear population using harvest samples

**DOI:** 10.1002/eap.70322

**Published:** 2026-10-05

**Authors:** Anthony Sévêque, Robert C. Lonsinger, Lisette P. Waits, Kristin E. Brzeski, Sarah L. Mayhew, D. Cody Norton, Caitlin N. Ott‐Conn, Tyler R. Petroelje, Anaïs K. Tallon, Maya Weissman, Dana J. Morin

**Affiliations:** ^1^ Department of Wildlife, Fisheries and Aquaculture, Forest and Wildlife Research Center Mississippi State University Mississippi State Mississippi USA; ^2^ Centre d'Etudes Biologiques de Chizé, UMR‐7372 CNRS‐La Rochelle Université Villiers‐en‐Bois France; ^3^ U.S. Geological Survey, Oklahoma Cooperative Fish and Wildlife Research Unit Oklahoma State University Stillwater Oklahoma USA; ^4^ Department of Fish and Wildlife Resources University of Idaho Moscow Idaho USA; ^5^ College of Forest Resources and Environment Science Michigan Technological University Houghton Michigan USA; ^6^ Wildlife Division, Michigan Department of Natural Resources Lansing Michigan USA; ^7^ Wildlife Division, Michigan Department of Natural Resources Marquette Michigan USA; ^8^ Animal GenoPhenomics Agroscope Posieux Switzerland

**Keywords:** abundance estimation, American black bear, close‐kin mark–recapture, genetics, harvest, kinship, terrestrial species

## Abstract

Close‐kin mark–recapture (CKMR) offers a promising alternative to traditional mark–recapture methods for estimating wildlife population parameters. CKMR estimates demographic parameters by identifying genetic relationships between individuals, effectively “recapturing” parental genotypes through their offspring's genetic profiles. One key advantage of CKMR over traditional methods is the ability to estimate abundance without recapture, facilitating the use of lethally collected biological samples. Here, we present an application of CKMR to a terrestrial species using samples collected from harvested individuals. We analyzed genetic data from 1767 American black bears (*Ursus americanus*) harvested over two consecutive years in Michigan's Upper Peninsula. Using Genotyping‐in‐Thousands by sequencing (GT‐seq), we identified parent‐offspring pairs and developed sex‐specific models tailored to black bear reproductive biology and management strategies. Female population estimates aligned with our expectations, based on prior knowledge of the population. In contrast, male population estimates deviated from expectations, potentially due to an unmodeled combination of factors pertaining to male reproductive biology and sampling bias. Our study not only demonstrates the viability of using harvest‐based CKMR for monitoring of terrestrial wildlife populations, but also highlights the need for careful model specification. By providing a novel method to estimate demographic parameters and population size, CKMR using harvest samples can inform management decisions and harvest quotas. This approach leverages existing management practices involving collection of genetic samples, such as mandatory harvest registrations and health monitoring programs. Our findings contribute to the growing body of CKMR applications and expand its use to terrestrial species management.

## INTRODUCTION

Adaptive wildlife management relies on effective population monitoring through recurrent and accurate estimates of animal abundance (Dolbeer, 1998; Morin et al., 2022). Mark–recapture (MR) methods have long been recognized as a powerful and widely used tool for estimating demographic parameters, population size, and trends (e.g., Karanth & Nichols, 1998; Royle et al., 2011). Advancements in genetic sampling expanded MR applications to species that cannot be easily captured and marked or visually identified as individuals (Lukacs & Burnham, 2005; Waits & Paetkau, 2005). However, the logistical and financial costs of repeated field‐based MR sampling (both invasive and noninvasive) can be costly, prohibiting adequate recurrent sampling over large spatial scales to detect population changes (Balme et al., 2009; Wegge et al., 2004).

Close‐kin mark‐recapture (CKMR) offers an alternative approach to estimate demographic parameters within a MR framework (Bravington, Skaug et al., 2016). Unlike traditional MR methods, CKMR does not require the recapture of the same individual. Instead, it relies on “recapturing” the genotype of a mature individual through the genotype of its offspring, either directly (the parent and the offspring are sampled; e.g., Bravington, Grewe et al., 2016) or indirectly (two siblings are sampled but not the parent; e.g., Hillary et al., 2018). Because recapture of the same individual is not required, CKMR can be used to monitor populations based on genetic samples obtained from lethal (e.g., harvest) and nonlethal (e.g., live trapping) sampling methods.

Collecting genetic samples from harvested animals is feasible for various species, particularly those subject to regulatory checks or tagging requirements. For example, in the United States, species such as bobcat (*Lynx rufus*) and river otter (*Lontra canadensis*) require export tags under the Convention of the International Trade in Endangered Species of Wild Fauna and Flora (CITES) for international trade (under federal regulations 50 C.F.R. § 23.69), providing opportunities for genetic sampling in states that enforce mandatory inspections. Additionally, management programs that include health monitoring of harvested species offer additional opportunities for genetic sample collection. Notable examples include the ongoing monitoring of wild boar (*Sus scrofa*) in several European countries (Rossi et al., 2023) and the surveillance of white‐tailed deer (*Odocoileus virginianus*) in North America for chronic wasting disease (Rivera et al., 2019).

To date, CKMR has primarily been applied to aquatic species (e.g., Bradford et al., 2018; Bravington, Grewe et al., 2016; Hillary et al., 2018; Ruzzante et al., 2019). Two applications have focused on terrestrial species (Christmas Island flying fox [*Pteropus natalis*]; Lloyd‐Jones et al., 2023, and boreal caribou [*Rangifer tarandus*]; Merriell et al., 2024), both using non‐lethal sampling due to the species' conservation status. More recently, CKMR has been applied to a harvested marine mammal, the bearded seal (*Erignathus barbatus*), using genetic samples collected from subsistence harvests of Native communities (Taras et al., 2024). In this paper, we present an application of CKMR to a terrestrial species using lethal samples collected at harvest. We used data collected from 1767 American black bears (*Ursus americanus*) harvested over two consecutive years in the Upper Peninsula of Michigan, USA, and used Genotyping‐in‐Thousands by sequencing (GT‐seq; Campbell et al., 2015) to identify parent‐offspring pairs (POPs). We developed sex‐specific models tailored to the respective reproductive biology and management strategies of male and female black bears. We evaluated the suitability of CKMR models to monitor black bears in Michigan and the implications of using samples from harvest in CKMR studies.

## METHODS

### Study area and sampling

Michigan's Upper Peninsula is heavily forested, with approximately 84% of its total land area (42,420 km^2^) characterized as forests dominated by northern hardwoods (e.g., maples [*Acer* spp.], oaks [*Quercus* spp.]), aspens (*Populus* spp.), and conifers (e.g., pines [*Pinus* spp.], spruces [*Picea* spp.]). The peninsula is bounded by Lake Superior to the north, Lake Michigan and Lake Huron to the south‐east, and a terrestrial border with Wisconsin to the south‐west. American black bear harvest is an important recreational activity in Michigan, with annual fall harvests ranging from 1500 to 2000 bears. Cubs (<1 year old [yo]) and females with cubs are protected and cannot be legally harvested. Within 72 h of harvest, hunters are required to take a harvested bear's head and pelt, or the entire animal, to be registered by the Michigan Department of Natural Resources (MI DNR).

The MI DNR currently uses a statistical catch‐at‐age model (SCAA) to monitor the black bear population and inform harvest quotas (Gove et al., 2002; Mayhew, 2019). This model uses age‐at‐harvest data and measures of hunter effort, both of which are routinely collected by management agencies, to estimate demographic parameters. However, SCAA models are highly sensitive to initial values of population size and require periodic independent estimates of abundance for calibration (Mayhew, 2019). The MI DNR systematically collects a premolar tooth from harvested bears during registration to determine their age through cementum annuli (Coy & Garshelis, 1992; Willey, 1974), which in turn informs the SCAA model. These teeth also contain high‐quality genetic information in the pulp (Higgins & Austin, 2013), which can potentially be used to estimate population size with CKMR.

### Genotyping

#### DNA extraction

We extracted DNA from tissue (rib muscle or tongue, 2021 only) and premolar tooth lysate (2021 and 2022) obtained from 2021 (*N* = 1421 bears) and 2022 (*N* = 1977 bears) fall harvests in Michigan's Upper Peninsula (Figure 1). We extracted DNA from tissue samples with a Qiagen DNeasy Blood and Tissue extraction kit (Qiagen, Germantown, MD, USA), following the manufacturer's instructions. For tooth lysate, teeth were extracted from harvested bears for aging. Prior to aging, we incubated whole teeth overnight in lysis buffer with proteinase K to break down any residual gum and pulp tissue. The teeth were then removed, and the resulting lysate was used for DNA extraction with the MagMAX CORE Nucleic Acid Purification Kit on a KingFisher Flex Purification platform (Thermo Fisher Scientific, Waltham, MA, USA). We used the 2021 tissue samples to design the GT‐seq panel and then applied this panel to the combined 2021–2022 tooth samples to establish kinship relationships and inform the CKMR model.

**FIGURE 1:**
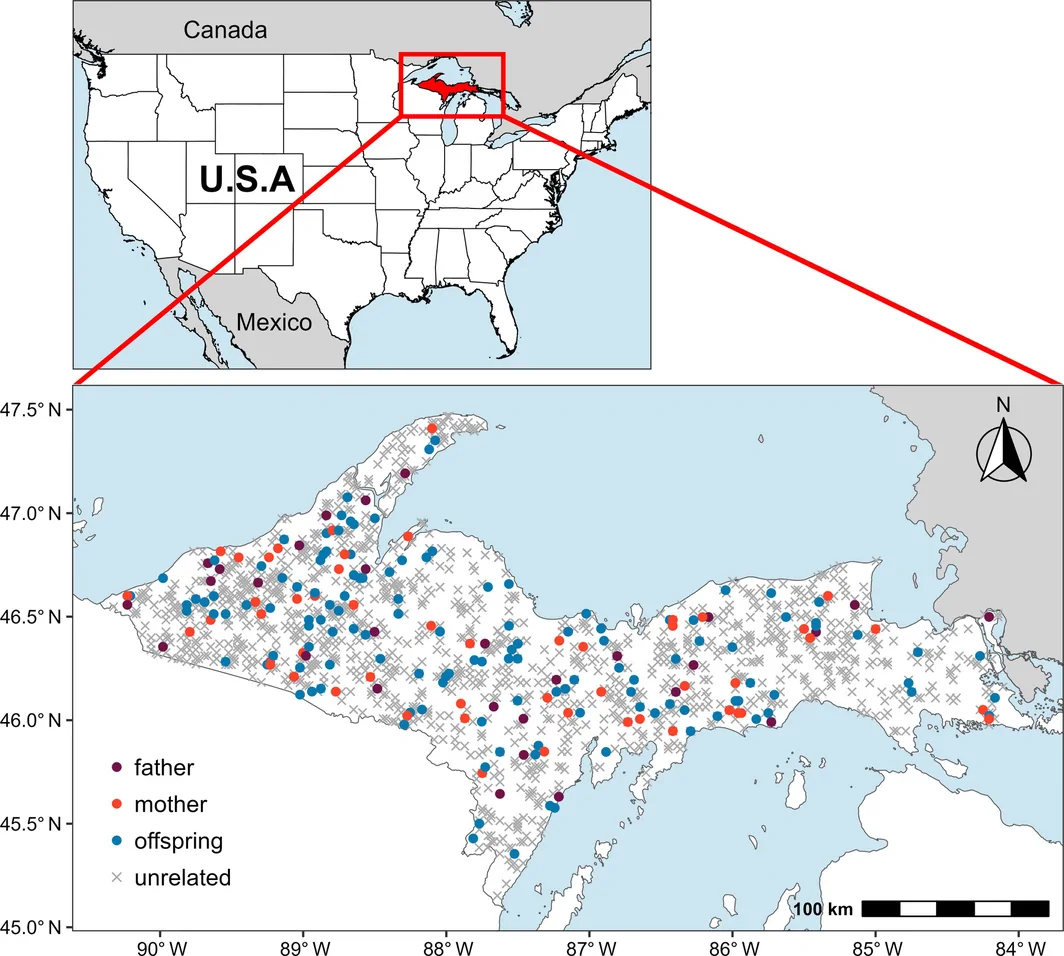
Map of samples collected from 1767 harvested American black bear (*Ursus americanus*) in 2021 and 2022 and retained for close‐kin mark–recapture analysis in the Upper Peninsula of Michigan, USA (2021–2022). Sampled bears were genotyped using Genotyping‐in‐Thousands by sequencing (GT‐seq) to identify mother‐ and father‐offspring pairs (color‐coded). One individual was both a father and an offspring (colored as a father for simplicity). Unrelated refers to any relationship that is not a parent‐offspring pair.

#### GT‐seq panel design

To establish a GT‐seq single nucleotide polymorphisms (SNPs) panel for Michigan black bears from which we could cost‐effectively genotype thousands of harvested bears, we first generated restriction site‐associated DNA sequencing (RAD‐seq) data from the population following methods outlined in Ali et al. (2016). This was done to discover polymorphisms prevalent within the population, where we selected 48 individuals that were representative of the entire study area. Samples were digested by the SbfI restriction enzyme and ligated eight base pairs (bp) barcoded, biotinylated adapters. After pooling and shearing to 400 bp with a Covaris LE220, libraries were enriched using a streptavidin bead‐binding assay and prepared with the NEBNext Ultra DNA Library Prep Kit (New England Biolabs, Ipswich, MA, USA). We conducted a bead‐size selection for 300–400‐bp fragments then standardized and diluted the libraries to 10 nM for sequencing on an Illumina NovaSeq 6000 flow cell. The raw RAD‐seq reads were processed using STACKS v2.3 (Catchen et al., 2013). We removed PCR duplicates and low‐quality reads, mapped to a publicly available black bear genome (National Center for Biotechnology Information accession GCF_020975775.1) using BWA‐MEM v0.7.12 (Li, 2013; Li & Durbin, 2009) and filtered with Samtools v1.9 (Li et al., 2009). Samtools also converted SAM to sorted BAM files, with SNP variants detected by STACKS v2.3 using the program gstacks.

These genotypes were then used to identify SNPs for panel development. We collaborated with GTseek LLC (https://gtseek.com/) to select SNPs and design primers that would work in a large, polymerase chain reaction (PCR) with >200 loci. Given that the panel was intended for kinship analysis, we focused on high minor allele frequency SNPs. These were selected from a variant call format (VCF) file generated using the RAD‐seq data containing over 9000 SNP loci as candidates. A set of 322 loci with minor allele frequencies between 0.20 and 0.45 were selected as targets for the GT‐seq panel. The VCF file was also used to variant‐mask the genome assembly using ambiguity codes for any SNP with an allele frequency >5% among the samples. Flanking sequence surrounding target SNP loci was gathered from the variant‐masked version of the genome assembly using a custom perl script. These data were then converted to fasta format for primer design using GTseek LLC's custom primer design services for multiplex PCR.

The proprietary pipeline of GTseek LLC uses a series of python and perl scripts to design many sets of primers per target and makes comparisons between the primer sets with the objective of producing a single primer set for multiplex PCR (Campbell et al., 2015). Each designed primer is tagged with its proper Illumina sequencing R1 or R2 site and checked for stable hairpin structures. Primers with hairpin structures with predicted melt temperatures above 50°C were rejected. Primers were also tested for likely heterodimers against any primers already in the primer set. Predicted heterodimers with melt temperatures above 50°C were rejected, and the algorithm moved on to the next primer for that locus. When no compatible primer sets were found for a locus, that locus failed to design a proper primer set and the algorithm moved on to the next locus. As a final filtering step, any primers with nested primer sites within any other amplicon were rejected from the panel to avoid concatemers in multiplex PCR. In addition, we included four sex‐linked markers to confirm field sex identification. We pruned the initial 322 loci to 280 loci, including sex‐linked markers for sex identification, through an iterative process of sequencing and removing loci that produced large amounts of “artifact” reads within the sequencing data or that did not have the expected SNP variant (Campbell et al., 2015).

#### GT‐seq library preparation

We employed a two‐step PCR approach following the methodology described by Campbell et al. (2015). In the first multiplex PCR reaction (PCR1), all 280 primer pairs were combined in a single reaction. Each well contained 3.5 μL of 2X Qiagen Multiplex PCR Plus Master Mix, 1.5 μL of a primer master mix (0.25 μM per primer), and 2.0 μL of template DNA. The thermal cycling conditions included an initial denaturation at 95°C for 15 min, followed by five cycles of 95°C for 30 s, 57°C for 30 s, and 72°C for 2 min. To optimize annealing, the temperature was gradually reduced with a slow cooling ramp rate (~0.1–0.3°C per second). This was followed by 13 additional cycles at 95°C for 30 s, 65°C for 30 s, and 72°C for 30 s.

After PCR1, the amplicons were diluted 20‐fold and used as the template for the second‐round PCR (PCR2), which incorporated indexing primers. Each well contained 5 μL of 2X Qiagen Multiplex PCR Plus Master Mix, 1 μL (10 μM) of a forward indexing primer, 1 μL (10 μM) of a reverse indexing primer, and 3 μL of the diluted PCR1 product. PCR2 thermal cycling conditions consisted of an initial denaturation at 95°C for 15 min, followed by 10 cycles of 95°C for 10 s, 65°C for 30 s, and 72°C for 30 s, with a final extension at 72°C for 5 min. Following amplification, PCR2 products were purified and normalized using the Just‐A‐Plate 96 PCR Purification and Normalization Kit (Charm Biotech), following the manufacturer's protocol. The resulting samples were pooled by combining 5 μL from each well, and the final library concentration was measured using Qubit 3.0 fluorometry (Thermo Fisher Scientific, Waltham, MA, USA). The final pooled libraries were quantified and sequenced on an Illumina NextSeq 2000 using a P2 2 × 150 nt kit, with a 1% phiX spike‐in and a final loading concentration of 800 pM, utilizing on‐board dilution and denaturation. Additionally, 600 μL of a 0.6 μM custom primer, diluted in HT1 hybridization buffer, was loaded onto the sequencing cartridge to facilitate i5 index capture (Hervey et al., 2025).

#### Data filtering and assessing confidence in kinship assignment

Prior to conducting kinship analyses, we performed a series of initial data‐cleaning steps to ensure data quality for downstream analyses (Appendix S1: Table S1). First, we pruned samples based on SNP coverage (reads at ≥50% of SNPs), confidence in assigning genetic sex (consensus among at least 3/5 sex markers), and noisiness of reads (individual fuzziness index [IFI] >2, an indication of cross‐contamination or unreliability). Sequencing success exhibited a bimodal distribution, with samples either amplifying consistently across loci or failing broadly (Appendix S1: Figure S1); thus, the 50% SNP coverage threshold functioned to remove samples that did not sequence reliably. We determined that these filtering steps did not introduce any biases in geography (Figure 1), sex, or age. Furthermore, we applied a coverage‐based filter to the SNPs, retaining only SNPs present in over 50% of the samples, as loci with high missingness would not contribute reliable information to kinship inference. The distribution of SNP performance for both genotyping and sex markers is shown in Appendix S1: Figure S1. We found no difference in quality control success between tissue and tooth extracts, with most variability instead arising from differences among GT‐seq runs and sample preparations. This process resulted in a final set of 211 SNPs for genotypes and 5 for sexing (total 216 SNPs) in our downstream analyses.

We used the CKMRsim R Package (Anderson, 2024) to assess the robustness of our nuclear DNA data in discerning relationships. All analyses were performed using the R statistical software version 4.3.2 (R Core Team, 2022). CKMRsim simulates genotypes with known relationships (i.e., parent‐offspring, full siblings, half‐siblings, and unrelated) based on the provided sequence data. Then, CKMRsim calculates the likelihood that any pair of simulated genotypes exhibits each of these relationship categories. Abundance estimates derived from CKMR relate to the number of reproductive adults that were alive when the juveniles were born, rather than when individuals were sampled, allowing integration of multiyear harvest data. We also simulated the effect of missing data on error rates, to determine the minimum number of SNPs needed to confidently assign relationships (Appendix S1: Figure S2). We calculated both false positive and false negative rates when simulating genotypes using 211 (or all autosomal loci), 190, 165, 158, 106, and 53 SNPs, selected randomly. We compared the ability to discern different focal relationships from unrelated individuals: POPs, full‐sibling pairs, and half‐sibling pairs (HSPs). False positive rates represent the probability of misclassifying an unrelated pair as the focal relationship. False negative rates represent the probability of misclassifying a pair with the focal relationship as being unrelated. Following CKMRsim recommendations, we aimed for a threshold for an acceptable false positive rate at least 10× smaller than the reciprocal of the number of comparisons (Anderson, 2024).

#### Pedigree construction

Finally, we constructed a pedigree among the 1767 high‐quality samples (2021 and 2022 combined) using the Sequoia R Package (Huisman, 2017). The POPs inferred from Sequoia were used as the observed kin pairs in the CKMR models described below, whereas CKMRsim was used to evaluate the expected reliability of relationship assignment from the SNP panel. Sequoia is a hill‐climbing algorithm that maximizes the overall likelihood that the total pedigree is correct. First, for each individual, Sequoia determines the list of possible parents using age‐difference based prior probability ratios. Here, we defined the age prior as potential parents reaching sexual maturity at 3 yo, with the upper limit for reproduction set at the maximum age observed in our dataset. Error in age estimation via cementum annuli is minimal for black bears from Michigan (Mayhew, 2019), and we assumed aging to have been done without error for the remainder of the analysis. Sequoia's pedigree reconstruction works in several steps. First, for each potential POP, Sequoia calculates the likelihood of seven possible relationships, including parent‐offspring. Then, using these pairwise likelihoods, Sequoia determines the most probable parents for each individual, if any exist in the dataset. After parentage assignment, Sequoia applies a similar process to identify likely sibling relationships among individuals. Finally, Sequoia employs a hill‐climbing algorithm, repeatedly refining these relationship assignments. This iterative process continues until the overall pedigree likelihood reaches its maximum value, allowing Sequoia to construct a comprehensive multigenerational pedigree that best fits the genetic data provided.

Sequoia generates a pedigree as its primary output, which can be reformatted to produce a comprehensive list of likely pairs. For each pair, we recorded the identity of both individuals, their most likely relationship, and the log likelihood ratio (LLR) supporting that focal relationship. The LLR is calculated as the logarithm of the ratio between the likelihood of the stated relationship and the likelihood of the next most probable relationship. When the LLR is positive, the stated relationship is more likely than any alternative. Conversely, a negative LLR suggests that another relationship might be more probable. While a negative LLR implies lower confidence in the assigned relationship, Sequoia may still include these pairs in the final pedigree if they contribute to maximizing the overall pedigree likelihood. To assess the reliability of our pedigree, we examined four distinct models in Sequoia, each with different primary settings (Appendix S1: Table S2, Figure S3).

### CKMR models

#### General parent‐offspring probabilities

We define $P\left[K_{\mathrm{ij}}=\mathrm{POP}|z_{i},z_{j}\right]$ as the probability that individual *i* is the parent of individual *j*, conditional upon individual covariates $z_{i}$ and $z_{j}$. Assuming that the sampling probability of *i* and *j* are independent, then $P\left[K_{\mathrm{ij}}=\mathrm{POP}|z_{i},z_{j}\right]$ depends on the expected reproductive output of the parent ($R_{i}$) at the place ($x_{j}$) and time ($b_{j}$) that *j* was born, relative to the total expected reproductive output of all parents ($R_{T}$) at the same place and time ($x_{j},b_{j}$).

(1) $$P\left[K_{\mathrm{ij}}=\mathrm{POP}|z_{i},z_{j}\right]=\frac{E\left[R_{i}\left(x_{j},b_{j}\right)|z_{i}\right]}{E\left[R_{T}\left(x_{j},b_{j}\right)\right]}.$$

Considering the size of Michigan's Upper Peninsula and the natal dispersal patterns of black bears in the region, including female philopatry (Moore et al., 2014), we expected to see spatial genetic differentiation within the population (i.e., the likelihood that two individuals are related is influenced by their spatial proximity). When this differentiation is coupled with spatially varying sampling probabilities (spatial differences in harvest), CKMR estimates of population size can become biased (Conn et al., 2020; Davies et al., 2017), unless dispersal patterns are accounted for in the kinship probabilities (Sévêque et al., 2025). In the case of black bear harvest in Michigan, spatial bias in sampling does not appear to be a significant issue. The higher sample size observed in the western part of the peninsula corresponds to a greater population density in that area (Fowler, 2021; Kautz et al., 2019; Norton et al., 2018). Consequently, we implemented a panmictic CKMR model and removed $x_{j}$ from the kinship probabilities.

Furthermore, because sampling is lethal, we do not need to account for the survival probability of the parent between its sampling year ($y_{i}$) and the birth year of its potential offspring ($b_{j}$). If $y_{i}<b_{j}$, then $P\left[K_{\mathrm{ij}}=\mathrm{POP}|z_{i},z_{j}\right]=0$. Given the two aforementioned considerations (panmictic population and lethal sampling), we can express the expected relative reproductive output term from Equation (1) as a practical numerical term by focusing only on the parameters and covariates that affect the expected reproductive output of parents.

#### Female‐focused model

Age of primiparity of female black bears harvested between 1994 and 2015 was estimated with cementum annuli from premolar teeth (Allen et al., 2017; Coy & Garshelis, 1992). With very few exceptions, females reproduced for the first time between 3 and 6 yo (Figure 2). The proportion of sexually mature females in this segment of the population was taken into account when estimating the total expected reproductive output (e.g., only 15% of all unrelated pairwise comparisons involving a 3‐yo female adult were retained as potential mother‐offspring (MO) pairs, while the rest was discarded under the assumption that the female was not yet sexually mature). Data from collared black bears and examination of reproductive tracts of harvested females in Michigan from 1993 to 2011 found no significant correlation between a mother's age and the size of her litter (Mayhew, 2019). In addition, the reproductive output of female black bears may be primarily determined by the abundance and quality of available food resources in their habitat and the overall physical condition of females (Costello et al., 2003; McDonald & Fuller, 2001); thus, we assumed that mature female black bears, regardless of their age, had a constant expected reproductive output. In addition, we removed any pairwise comparisons in which the mature female was sampled the same year the potential offspring was born (implying the possible harvest of a mother with her cub‐of‐the‐year, which was prohibited in Michigan).

**FIGURE 2:**
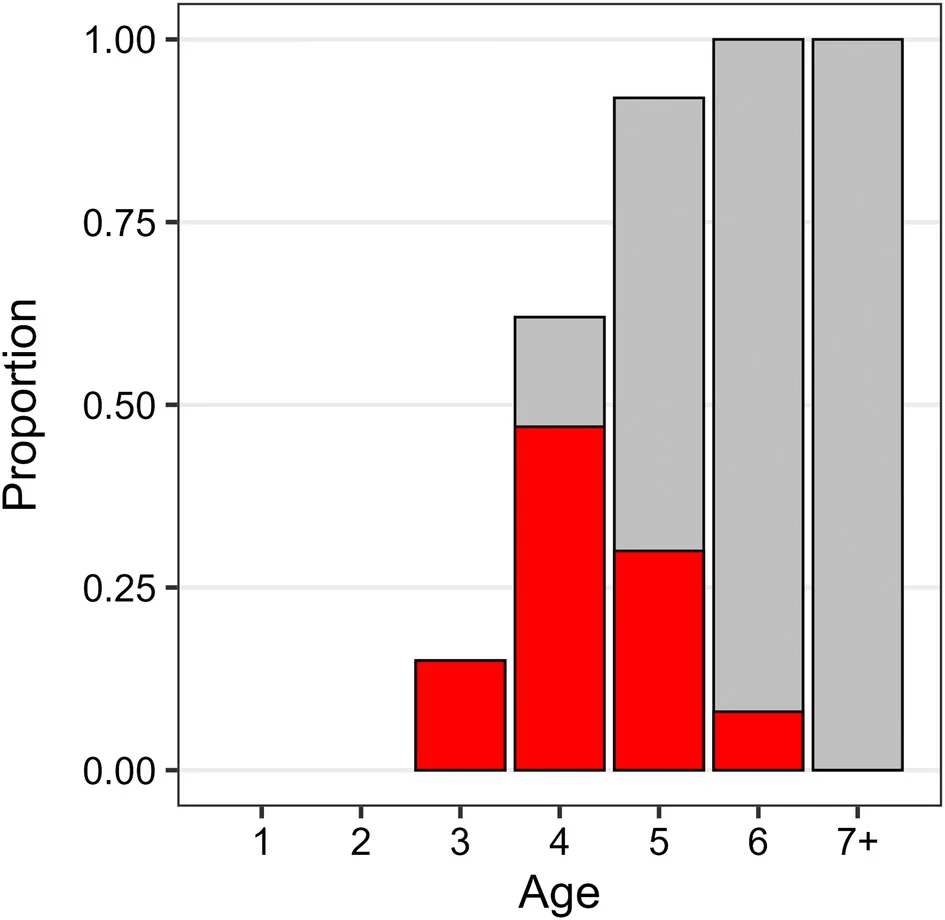
Distribution of age at sexual maturity and proportion of reproductive individuals of female American black bears (*Ursus americanus*) in Michigan, USA, 1994–2015. Red bars indicate the proportion of individuals that reached sexual maturity at a given age, whereas gray bars indicate the total proportion of females that were sexually mature at a given age.

A characteristic feature of black bear reproductive biology is intermittent breeding, wherein females typically adhere to a biennial reproductive cycle (i.e., mothers remain focused on maternal care and do not engage in reproduction the year immediately following the birth of cubs). There is no requirement to account for intermittent breeding in the expression of the MO probabilities, provided that two conditions are met. In the case of black bears, the first condition requires that all mature females in the population strictly follow a biennial breeding cycle. This assumption will be violated when a substantial proportion of females consistently reproduce in back‐to‐back years (e.g., if total litter loss is common and disrupts the normal breeding rhythm), or when years of particularly poor natural food availability cause females to fail to produce a litter (i.e., a 3‐year gap between successful reproduction events). The second condition requires that approximately half of the mature female population breeds 1 year, while the other half remains nonreproductive. In the following year, the nonreproductive females become the breeding cohort and the previous year's breeders do not reproduce (in the case of biennial breeding cycle). This assumption may not hold true in populations where mature females synchronize their reproductive efforts corresponding with masting events, such as the temporary synchrony of black bear reproduction with the beech (*Fagus grandifolia*) masting cycle in Maine, USA (McLaughlin et al., 1994). When the two conditions are met, the expected reproductive output of a female in a given year is reduced by half (50% chance of being in breeding phase) and the total expected reproductive output pertains to half of the female population (the reproductive portion). As a result, the expected relative reproductive output remains constant.

In Michigan's Upper Peninsula, total litter loss among monitored black bears was rare (Norton et al., 2018), and the massive mast failures that would lead to failed reproduction and synchrony were either infrequent or bears could find alternative food sources to maintain biennial breeding (Mayhew, 2019). Therefore, we believe that the two conditions were met for the Upper Peninsula Michigan population, and we did not incorporate intermittent breeding in the MO kinship probabilities. In addition, females actively caring for cubs‐of‐the‐year were protected from harvest in Michigan (and, by extension, from sampling). However, because we used harvest data from two consecutive years, we assumed that our sample represented the full pool of potential mothers. This assumption would not hold in a biennial breeding system if sampling occurred in a single year, since only one reproductive cohort (roughly half the population) would be available for capture at that time.

Given constant fecundity, lethal sampling, panmictic population, random sampling, and known ages, we can express the probability that two animals are related as a MO pair as follows:

(2) $$P\left[K_{\mathrm{ij}}=\mathrm{MO}|b_{i},b_{j},y_{i}\right]=\left\{\begin{array}{cc} \frac{1}{N_{♀,b_{j}}} & \text{if}\;i\;\text{is alive and mature}\;\mathrm{at}\;b_{j}\;\\ 0 & \text{otherwise} \end{array}\right.,$$

where $N_{♀,b_{j}}$ refers to the abundance of mature females the year *j* was born.

Equation (2) informs on adult abundance the year *j* was born ($b_{j}$), which is highly variable in our dataset (2000–2021). Obtaining annual independent population estimates is possible but can be inefficient and lead to yearly estimates with poor precision. Instead, we integrated kinship probabilities into a population dynamics model that includes a term for average annual population rate of increase (*r*) over the period between the latest and earliest birth years of any *j* in the samples (e.g., Lloyd‐Jones et al., 2023; Patterson et al., 2022). The base population dynamics model for females is

(3) $$N_{♀,t}=N_{♀,2020}\times \exp^{\left[r_{♀}\times \left(t-2020\right)\right]},$$

where $N_{♀,t}$ is the adult female population size in an arbitrary unit of time *t* and $r_{♀}$ is the female annual population rate of increase. Here, we chose to derive $N_{♀,t}$ from the most frequent $b_{j}$ among offspring in the samples (2020). Alternatively, one could derive $N_{♀,t}$ from $t_{0}$, where $t_{0}$ is the initial year of modeling (i.e., the oldest $b_{j}$ in the samples), which should yield similar estimates of population parameters.

#### Male‐focused models

Knowledge on male black bear reproductive biology is scarce in comparison to females. To address this gap, we explored various biologically plausible scenarios and compared the estimates of male population size. In addition, black bears exhibit delayed implantation (or embryonic diapause), a biological mechanism where fertilization is postponed after mating. This means that a male that breeds in spring could be harvested during fall harvest, and yet still be the father of cubs born in den early in the following year. In this context, the expected relative reproductive output term (Equation 1) is not informed by expected reproductive output and adult abundance the year *j* was born ($b_{j}$) but rather aligns with the prior year ($b_{j}-1$) when the actual reproduction took place. We considered two scenarios for the age at sexual maturity in males: one where they reach maturity at 3 yo, and another at 4 yo. However, it is important (especially for males) to distinguish between the age of sexual maturity and the age at first reproduction. While males may be sexually mature by 3 yo, their chances of successfully mating with a female at this age could be substantially diminished due to competition with older males, as has been observed in several bear species (Kovach & Powell, 2003; Richardson et al., 2020; Zedrosser et al., 2007). Therefore, we implemented two scenarios of variation in expected reproductive output in our models. The first scenario was similar to the approach used for females and assumed a constant expected reproductive output for all mature males, with the probability that two individuals are related as a father‐offspring (FO) pair defined as

(4) $$P\left[K_{\mathrm{ij}}=\mathrm{FO}|b_{i},b_{j},y_{i}\right]=\left\{\begin{array}{cc} \frac{1}{N_{♂,b_{j}-1}} & \text{if}\;i\;\text{is alive and mature}\;\mathrm{at}\;b_{j-1}\\ 0 & \text{otherwise} \end{array}\right..$$

In contrast, the second scenario incorporated age‐dependent variations in expected reproductive output, based on patterns observed by Costello et al. (2009). Although there is no requirement for all individuals to have equal reproductive output, variations in lifetime reproductive success can bias CKMR estimates based on POPs when the probability of sampling an adult is dependent on the number of its offspring sampled (Bravington, Skaug et al., 2016; Sévêque et al., 2024). We have no way of detecting within‐age variance in male reproductive success in our population, even though it is likely stronger than variation among age (Waples, 2023), nor can we directly detect fecundity‐related sampling bias. However, we can incorporate age‐dependent variations in reproductive success reported in other studies and evaluate potential age bias in our samples, given that the population's age distribution is known. This second scenario assumed a correlation between age, body size, and reproductive success, with reproductive success (or fecundity at age) reaching its peak at 11 yo when body size characteristics favorable to mating success are optimal (Figure 3).

**FIGURE 3:**
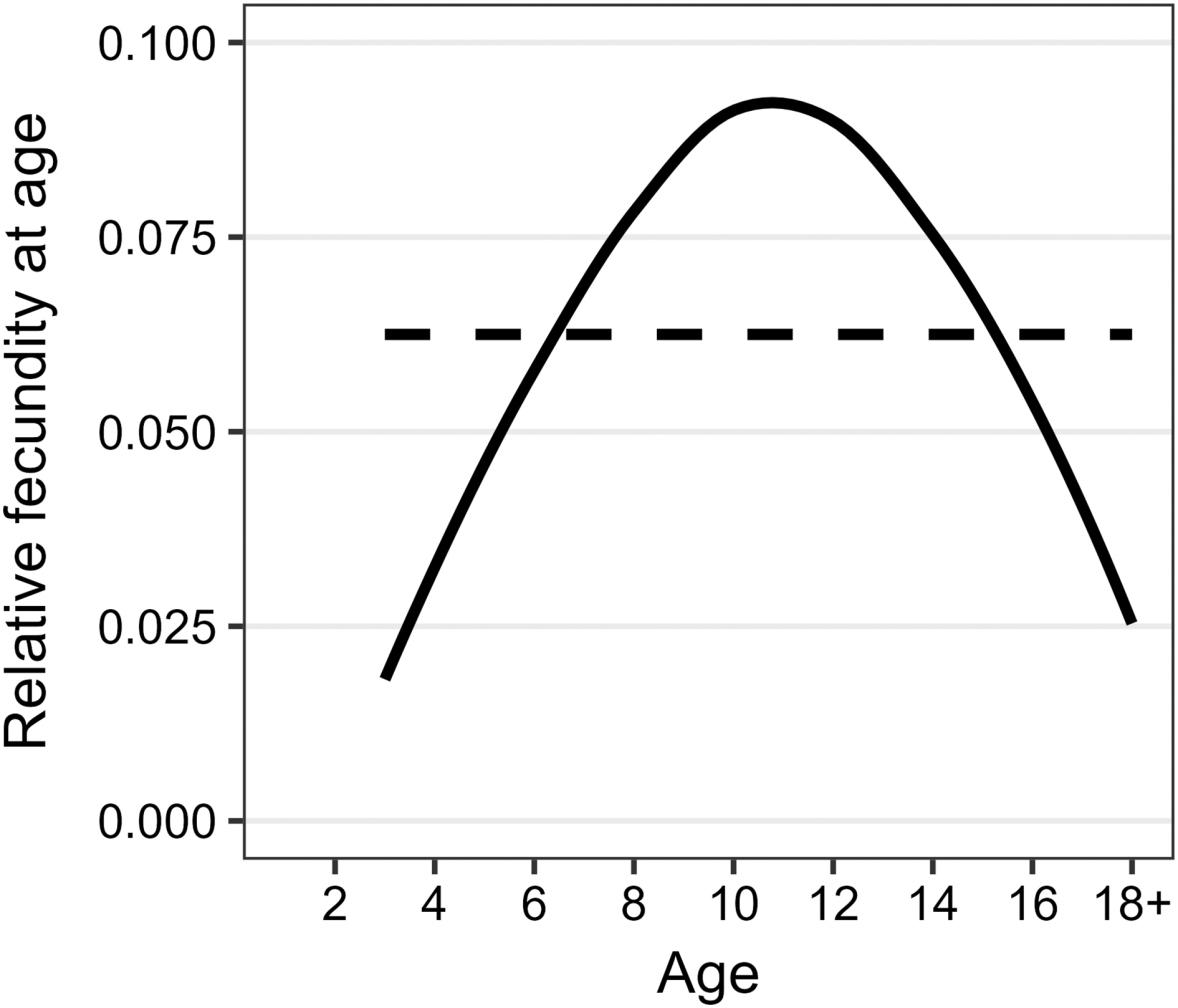
Relative fecundity at age of male American black bears (*Ursus americanus*) for two scenarios—constant expected reproductive output (dashed line) and age‐dependent expected reproductive output (solid line; following Costello et al. (2009). Values presented here were adjusted using a normalizing constant so that the sum of relative fecundity at age across all possible ages = 1.

For the male kinship probability in this scenario, the expected reproductive output of the father ($R_{i}$) is a function of his relative fecundity at age ($f_{a}$) at $b_{j}-1$. The total expected reproductive output ($R_{T}$) becomes the product of the number of mature males of each age and the corresponding relative fecundity at age, summed across all possible ages. This formulation requires having an explicit age‐structured population dynamics model for males. One option is to provide the model with an independent estimate of the age distribution of individuals if we suspect that the sampling may be biased and not representative of the overall age structure of the male population. Here, we used the age distribution from our samples directly, as it closely resembled the age distribution obtained independently from a previous SCAA model (Appendix S2: Figure S1; Mayhew, 2019) and appeared to be unbiased. The FO probability in this second scenario is

(5) $$P\left[K_{\mathrm{ij}}=\mathrm{FO}|b_{i},b_{j},y_{i}\right]=\frac{f_{a,b_{j}-1}}{\sum_{a}N_{♂a,b_{j}-1}\times f_{a}}.$$

The population dynamics model for males is

(6) $$N_{♂,t}=N_{♂,2020}\times \exp^{\left[r_{♂}\times \left(t-2020\right)\right]},$$

where $N_{♂,t}$ is the adult male population size at time *t* (obtained by summing all $N_{♂a,t}$) and $r_{♂}$ is the male annual population rate of increase.

#### Model implementation

We used Markov chain Monte Carlo (MCMC) methods within a Bayesian framework to fit the models. The posterior distributions of abundance (*N*) and population rate of increase (*r*) for each sex were obtained with the pseudo‐likelihood (presented as a single sex‐neutral equation here):

(7) $$\prod_{i<j}P\left(K_{\mathrm{ij}=\mathrm{POP}}|N_{2020},r,b_{i},b_{j},y_{i}\right),$$

formed from products across all pairs of sampled individuals (*i* and *j*) given that *i* was of the corresponding sex for each model and was born before *j*, combined with a scale prior on $N_{2020}$ and a uniform diffuse prior on *r* (−0.5 to 0.5). We used the mode of the posterior distribution as our estimate, and we compared the estimates of abundance obtained with CKMR to the latest SCAA estimates for the same year (Norton, 2025; Sévêque, 2024). We used four MCMC chains for each model with 100,000 iterations, a burn‐in of 25,000 observations, and a thinning rate of 10. We visually inspected trace plots to evaluate how well the chains were mixing and used effective sample size and a $\overset{`}{R}$ threshold of 1.01 for convergence diagnostics (Vehtari et al., 2021). We used the R package Nimble (de Valpine et al., 2017) to implement the MCMC sampling.

Equation (7) is a pseudo‐likelihood because the pairwise comparisons are not truly statistically independent (each offspring can only have one parent of each sex). Pseudo‐likelihoods can be valuable approximations of full likelihoods in scenarios where the sample size is small compared to the overall population size (sensu Bravington, Skaug et al., 2016). The sampling intensity used in this study (i.e., the proportion of the population that has been sampled) may be too high for this point to be true. If so, the use of a pseudo‐likelihood would likely result in a conservative estimation of uncertainty with overly wide confidence intervals (Conn, 2025; Merriell et al., 2024).

## RESULTS

The genetic analysis confirmed the reliability of GT‐seq in distinguishing first‐order kin pairs (Appendix S1: Figures S4 and S5). Following genetic quality control procedures, our final dataset contained 1749 samples (713 females and 1036 males), from which we identified 65 mother‐offspring pairs (54 individual mothers), 62 FO pairs when male sexual maturity was set at 3 yo (27 individual fathers), and 55 FO pairs (26 individual fathers) when male sexual maturity was set at 4 yo. The number of offspring assigned to fathers ranged from 1 to 7 in both scenarios. The age distribution of identified parents at year of birth of their offspring is illustrated in Appendix S2: Figure S2.

The estimated female adult population size in 2020 was 2915 individuals (95% credible intervals [CIs] 2258–3877; Figure 4A) and was comparable to the latest SCAA model estimate of the reproductive female population of 2468 individuals (Sévêque, 2024). The estimate of female population rate of increase indicated a slight but insignificant decrease in female population size ($\hat{r_{♀}}$= −0.066, 95% CI = −0.265–0.084; Figure 4B). Estimates of the male adult population size from CKMR were approximately 2× greater than estimates from the latest SCAA model (Sévêque, 2024), across all four scenarios examined (Figure 4A). When the age at first reproduction was set to 3 years, estimates ranged from 2370 (95% CI = 1865–3158) to 2904 individuals (95% CI = 2326–3950), compared to the most recent SCAA estimate of 1359 adult males. In scenarios where males were assumed to reach sexual maturity at 4 years, CKMR estimates ranged from 1728 (95% CI = 1371–2382) to 1963 individuals (95% CI = 1565–2723), again exceeding the latest SCAA estimate of 825 adult males. Incorporating age‐dependent fecundity into the model resulted in abundance estimates that were 15%–25% higher than those based on constant fecundity. Estimates of the population's rate of increase were positive when sexual maturity was set at 3 years and negative when set at 4 years (Figure 4B), although the 95% CI overlapped zero for all scenarios.

**FIGURE 4:**
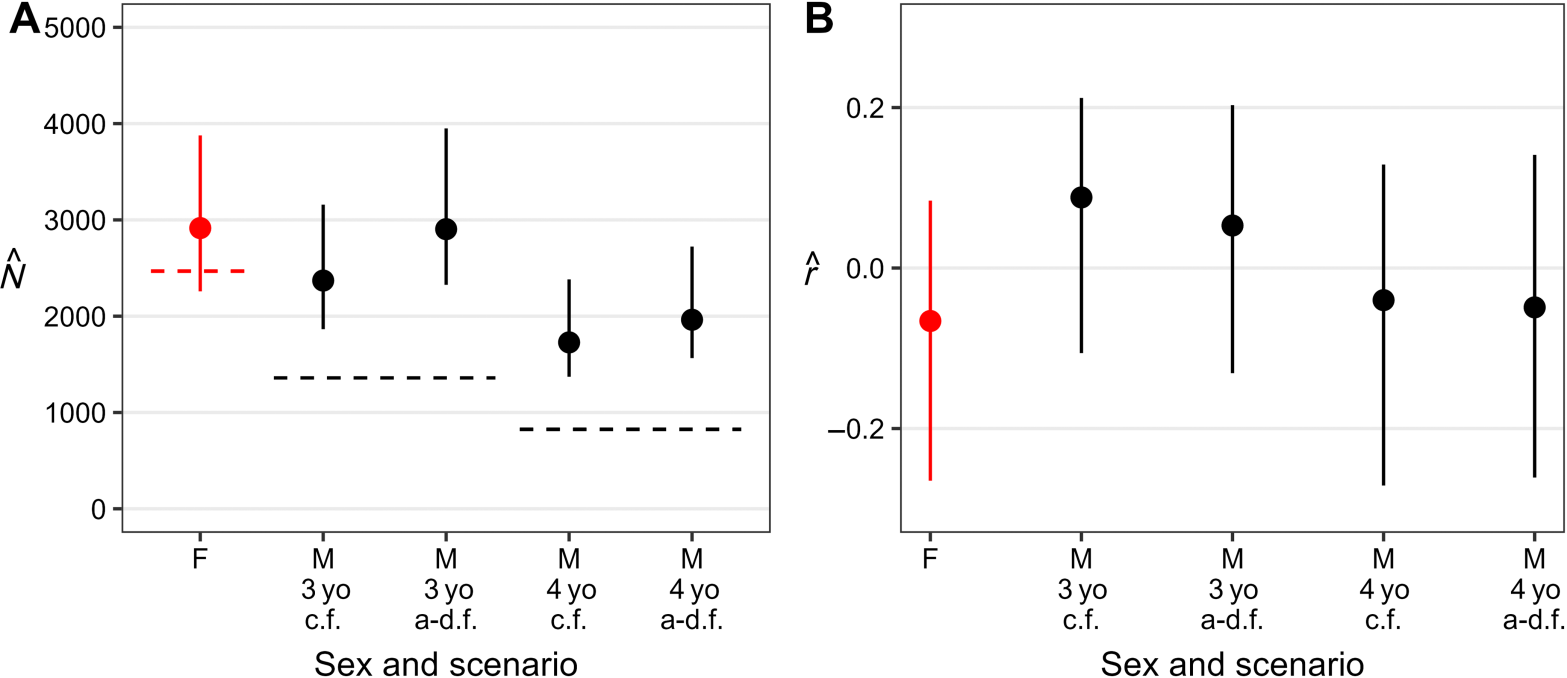
Close‐kin mark–recapture estimates of adult American black bear (*Ursus americanus*) population size $\hat{N}$ (A) and population rate of increase $\hat{r}$ (B) across two sexes (F, females in red; M, males in black) and four scenarios of age at first reproduction and variation in reproductive output for males: a‐d.f., age‐dependent fecundity; c.f., constant fecundity; yo, years old. Points show the mode of the posterior distributions; vertical bars show 95% credible intervals; horizontal dashed lines in (A) show the most recent abundance estimates from an independent statistical catch‐at‐age model.

## DISCUSSION

Our study demonstrates the applicability of CKMR to a model game species using harvest samples, paving the way for its development into a standard tool for estimating important population parameters such as abundance and trends. Our base female‐focused model performed well and produced estimates with sufficient precision for implementation in bear management (coefficient of variation = 14.6%; Pollock et al., 1990, Williams et al., 2002). The CKMR estimates suggested a slightly larger female population compared to the current SCAA estimates. The male‐focused models produced estimates that were roughly double those of the latest SCAA estimates. Although SCAA models themselves can be subject to bias (e.g., Conn & Diefenbach, 2007), our diagnostics suggest that the CKMR estimates for males are likely inaccurate, as extrapolating CKMR adult male abundance to all males aged 1+ resulted in an implausibly large population size and a heavily male‐biased sex ratio (noting that these diagnostics rely on the age structure defined by the SCAA model). These findings suggest that the male‐focused CKMR models implemented here may be overlooking significant factors affecting kinship probabilities, possibly linked to reproductive skewness or sampling probabilities.

Female‐focused models are likely to generate more accurate CKMR estimates compared to models centered on males, because the greater homogeneity and consistency in female reproductive biology align more closely with the assumptions of CKMR. In addition, estimating the abundance of adult females provides crucial insight into the effective breeding population size and population growth trends, which can be sufficient for monitoring population dynamics on their own (Caswell, 2001; but see Rankin & Kokko, 2007). Focusing on the female population is a well‐established approach of population monitoring (e.g., McMahon et al., 2005; McNay et al., 2022; Messmer et al., 2020), and we anticipate that this female‐centric approach will become increasingly common in CKMR as well (e.g., Lloyd‐Jones et al., 2023; Merriell et al., 2024).

Male abundance was most likely overestimated in all four models, indicating there were fewer FO pairs in the sample than expected. In Michigan's Upper Peninsula, success rate of bear harvest approximates 30% (Frawley, 2023), and is generally considered opportunistic. Thus, we assumed that there was no sampling selectivity related to fecundity (e.g., by targeting larger males), which could have otherwise biased CKMR estimates if unaccounted for. However, about 85% of bears tend to be harvested over bait in Michigan. Bait stations can be established from the start of the bear baiting period (31 days before the open season) through the end of the bear hunting season. Dominant, highly reproductive males may establish dominance around bait sites during the nocturnal period when hunting is prohibited and then retreat to safer areas during the diurnal period (Stillfried et al., 2015). This behavior could force subordinate, less reproductive bears to alter their feeding schedule and restrict access to bait sites for subordinate bears to higher risk diurnal periods, increasing harvest susceptibility. This scenario could suggest a “reverse fecundity dependent sampling” where less fertile mature bears are more likely to be harvested, resulting in fewer FO pairs in the samples. While this a posteriori explanation is somewhat anecdotal, the concept of despotic distribution, where dominant individuals exclude subordinate ones from anthropogenic food sources and high‐quality habitats, is well documented in black bears (e.g., Beckmann & Berger, 2003; Ditmer et al., 2016). Confirming this pattern would require a comparative male parentage analysis of harvest samples and a truly random sample of the population (e.g., via independent hair‐snare studies). If confirmed, a correction factor could be incorporated into the FO probabilities to account for this sampling bias.

The age of first reproduction did not strongly affect the overall results for males, as extrapolating mature male abundance from the 3 or 4 yo models to the total male population yielded similar estimates. This suggests that males reproducing at 3 yo may not be as rare as initially thought. Moreover, age‐dependent reproductive success had a moderate effect on the estimates (22.5% and 13.6% increase in the 3 and 4 yo models, respectively). This aligns with expectations that age‐dependent fecundity primarily affects HSPs probabilities and only influences POPs when combined with age‐dependent sampling probability (Sévêque et al., 2024). While such sampling effects do not appear to be strong for mature males in this study (Appendix S2: Figure S1), a weak age‐dependent sampling bias cannot be entirely ruled out. Finally, CKMR estimates of population rate of increase for both males and females suggested that the population size remained relatively stable; however, the estimates of population rate of increase had poor precision and did not allow for a clear determination of population trend. Regular, independent CKMR surveys of this population could help confirm or deny this pattern. The frequency of CKMR applications must be tailored to the demographic parameters of the monitored species and the specific needs of the monitoring program (e.g., every 5 years for integration into SCAA; Mayhew, 2019).

If obtaining an estimate of male population size is critical, CKMR models can also be fitted with HSPs (e.g., Davies et al., 2020; Hillary et al., 2018; Patterson et al., 2022). One of the key advantages of HSPs is that they do not require sampling the parent, thereby eliminating any effect of adult sampling bias. This feature could make targeting HSPs an effective strategy for detecting and correcting sampling bias in adults. However, HSPs probabilities are subject to more assumptions and potential sources of errors compared to POPs (Sévêque et al., 2024), especially for males who often exhibit reproductive skew (e.g., Lloyd‐Jones et al., 2023). Furthermore, genetically identifying second‐order relationships, such as HSPs, can be more challenging than first‐order relationships like POPs. First, the mating system of black bears is influenced not only by male‐to‐male competition and age of the male, but also by proximity‐based mating strategies aimed at reducing infanticide (Costello et al., 2009; Moore et al., 2015). This, combined with female philopatry (Costello, 2010; Moore et al., 2014) and the relatively high longevity of black bears, increases the proportion of full thiatic pairs (aunt/uncle‐niece/nephew pairs; “thiatic” denoting aunt or uncle) in the population, which are genetically indistinguishable from HSPs and cannot be filtered out based on age difference. The presence of these indistinguishable pairs can lead to significant biases in abundance estimates due to false positives (Conn, 2025). Second, differentiating between maternal and paternal HSPs requires genotyping mitochondrial DNA (mtDNA). Given that mtDNA is inherited exclusively from the mother, two half‐siblings that have different mtDNA profiles can be identified as a paternal HSP with full certainty. However, if they share the same mtDNA, they could be both maternal or paternal HSP (e.g., if both mothers are related and possess the same mtDNA). In terrestrial philopatric species with proximity‐based reproduction, distinguishing between maternal and paternal HSPs can prove challenging. To effectively address this issue, CKMR models would need to incorporate an explicit probability of being maternally or paternally related for each HSP, rather than assigning full certainty to one category or the other.

This study is the first application we know of CKMR to a terrestrial mammal using lethal samples obtained during harvest. Our models have provided estimates of female abundance and unveiled potential unknown insights into male harvest patterns. Despite encountering clear challenges, our study demonstrates that CKMR holds promise for regular and relatively inexpensive monitoring of harvested species. In contrast to traditional MR methods, CKMR does not require repeated capture sessions, eliminating costs associated with field trapping, tagging equipment, and extensive travel or field logistics. Sampling efforts can instead be concentrated on collecting genetic material from individuals already harvested as part of normal exploitation activities and performing laboratory‐based genetic analyses at relatively stable and predictable costs. However, CKMR estimates of abundance for the most recent sampling years (e.g., 2022 here) will exhibit low precision without growth parameters to link temporal dynamics, due to inherent lags in kin pair detection from recent cohorts. Even when including growth parameters, assuming a constant growth rate limits the ability to detect short‐term population changes. Given this limited responsiveness, CKMR may be better used as part of an integrated management strategy incorporating real‐time indices like harvest per unit effort or hunter success rates to inform annual management. Furthermore, future CKMR applications to harvest data should consider not only the reproductive biology of the species but also pay close attention to the sampling scheme, as harvest may not always be random with regard to reproductive success (e.g., trophy hunting; Coltman et al., 2003). It is also likely that many applications to terrestrial species with limited dispersal will need to implement spatially explicit CKMR models if harvest is spatially biased (Patterson et al., 2026; Sévêque et al., 2025). This study has laid the groundwork for future research in wildlife management by implementing a CKMR model that offers a cost‐effective and insightful approach to monitoring wild populations.

## AUTHOR CONTRIBUTIONS

Caitlin N. Ott‐Conn, Kristin E. Brzeski, Dana J. Morin, Robert C. Lonsinger, and Lisette P. Waits conceptualized the initial ideas; Dana J. Morin and Caitlin N. Ott‐Conn managed the project administration; Kristin E. Brzeski and Caitlin N. Ott‐Conn collected and processed the samples. Kristin E. Brzeski, Anaïs K. Tallon, and Maya Weissman designed the GT‐seq panel and conducted the kinship analysis. Sarah L. Mayhew, Cody Norton, and Tyler R. Petroelje provided the life history data required for the models; Anthony Sévêque and Dana J. Morin ran the formal analysis and interpreted the results; Anthony Sévêque led the writing of the manuscript with large contributions from Dana J. Morin, Robert C. Lonsinger, and Kristin E. Brzeski. All authors contributed critically to the drafts and gave final approval for publication.

## CONFLICT OF INTEREST STATEMENT

The authors declare no conflicts of interest.

## Supporting information


Appendix S1.

Appendix S2.

## Data Availability

Data and code for the bioinformatic pipeline (Weissman, 2026) are available on Zenodo at https://doi.org/10.5281/zenodo.19488498. Data and code for CKMR modeling (Sévêque, 2025) are available on Zenodo at https://doi.org/10.5281/zenodo.15364917.
