# Supplementary material for: Application of close‐kin mark–recapture to an American black bear population using harvest samples
Source: Ecol Appl. 2026 Oct 5;36(7):e70322. doi: 10.1002/eap.70322 (PMC13639227; doi:10.1002/eap.70322)
Supplement: Supplementary file 1 [file EAP-36-e70322-s001.pdf]

Appendix S1: Assessing confidence in pedigree relationships

Application of close-kin mark–recapture to an American black bear population using harvest samples

Anthony Sévêque, Robert C. Lonsinger, Lisette P. Waits, Kristin E. Brzeski, Sarah L. Mayhew, D. Cody Norton, Caitlin N. Ott-Conn, Tyler R. Petroelje, Anaïs K. Tallon, Maya Weissman & Dana J. Morin.

Ecological Applications

Any use of trade, firm or product names is for descriptive purposes only and does not imply endorsement by the U.S. Government.

Table S1. *Summary results from GTseq runs for 2,872 American black bears (Ursus americanus) harvested in the Upper Peninsula of Michigan during 2021–2022. SNPs = single nucleotide polymorphism.*

Total bears genotyped	2,872
2021 total bears genotyped	1,426
2022 total bears genotyped	1,396
Initial average % on-target reads	38.0%
Initial average % genotyped	73.4%
Bears with ≥ (quality control threshold)	1,767
Nuclear SNPs with ≥ 50% coverage	211
Mitochondrial SNPs with ≥ 50% coverage	33

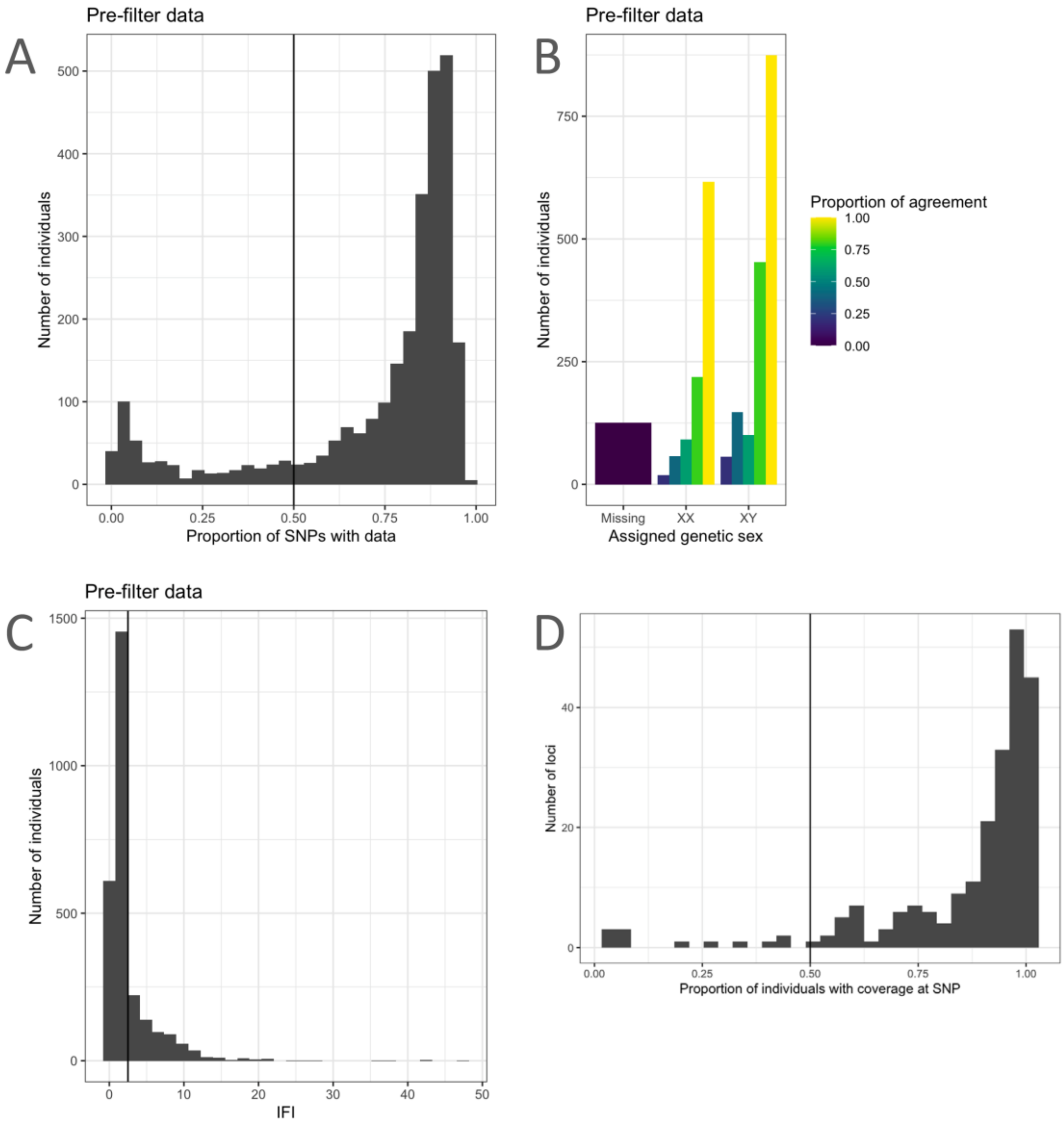

Figure S1. Initial data-quality filtering metrics: (A) Single nucleotide polymorphism (SNP)coverage per individual with a 50% cut off, (B) genetic sex marker concordance (females = XX; males = XY), (C) Individual fuzziness index (IFI), an indication of cross-contamination with <2 cut off, and (D) locus-level SNP missingness.

Assessing the robustness of the nuclear DNA data in discerning relationships.

We evaluated the power of our nuclear DNA data in discerning relationships first in the final set of 1,767 black bears using the CKMRsim R package (Anderson 2024). False positive and false negative rates were calculated using simulated genotypes. We varied the number of single nucleotide polymorphisms (SNPs) used (211, 190, 165, 156, 105, and 53) to simulate the impact of missing data and determine the minimum number of SNPs needed to confidently infer relationships. For all focal (numerator) relationships, the alternate (denominator) relationship used was "unrelated". False positive rates are the probability of misidentifying a pair whose true relationship is unrelated as the focal relationship. The threshold for an acceptable false positive rate is that it must be at least 10 times smaller than the reciprocal of the number of comparisons, which is shown as a horizontal black line. False negative rates represent the probability of misclassifying a pair with the focal relationship as being unrelated.

When discerning full-sibling relationships (FS), we had sufficiently high confidence in our ability to assign relationships when using 158 (75%) or more SNPs (Figure S1). Our panel had low confidence in assigning half-sibling relationships (HS), even when using all SNPs. For parent-offspring pairs (PO), our panel retained an acceptable false positive rate when using as few as 106 (50%) SNPs.

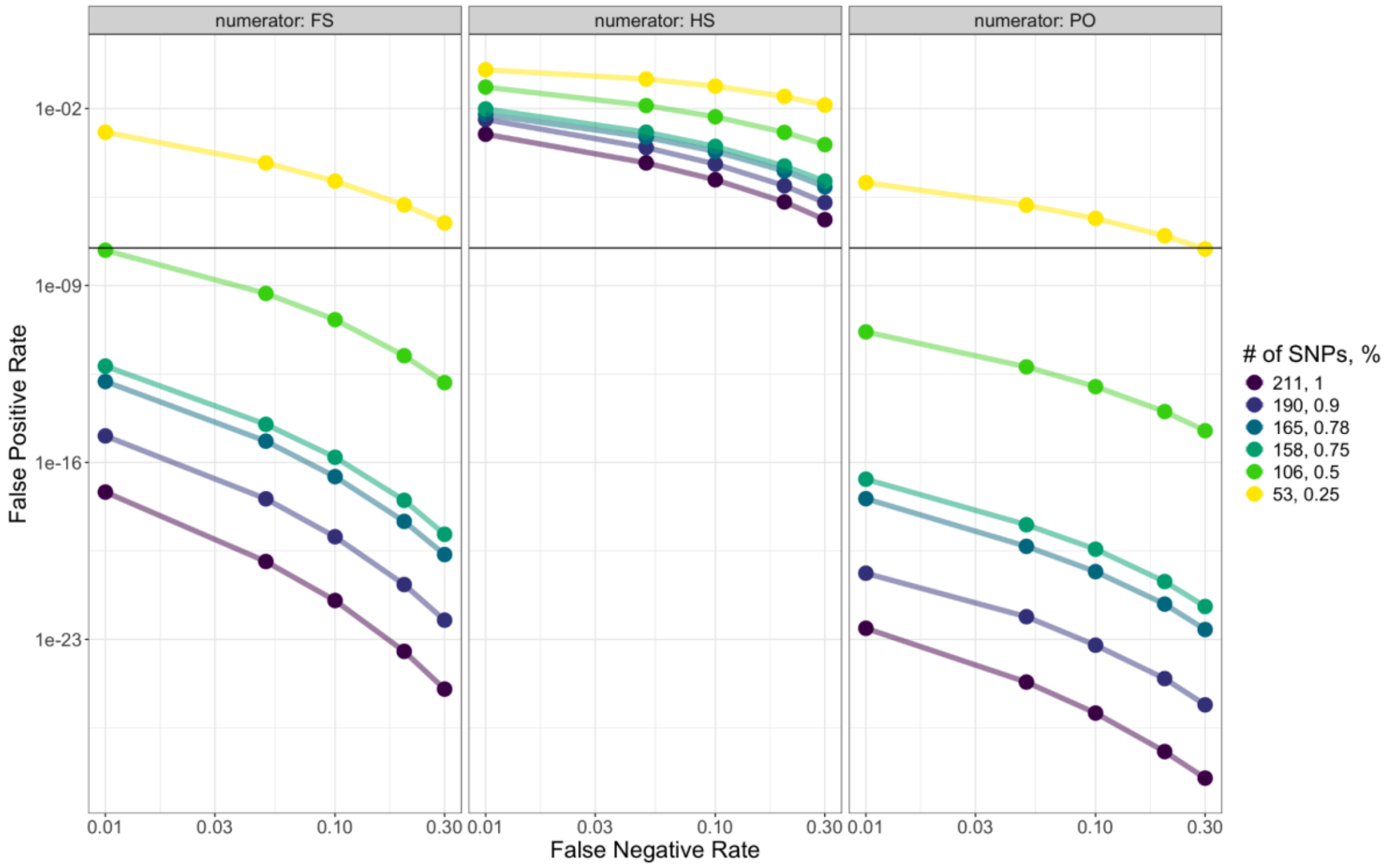

Figure S2. *False positive and false negative rates with varying number of SNPs used for pairwise relationship inference in three relationship types (full-sibling FS; half-sibling HS; parent-offspring PO).*

Assessing the reliability of the pedigrees obtained in four distinct models in Sequoia software.

Sequoia software (Huisman 2017) uses two primary settings: the age prior and the type of pedigree constructed (Parent-Offspring only (PO) or full). We tested the sensitivity of our results to changing these settings by constructing four pedigrees: using age priors and constructing a full pedigree, using age priors and constructing a PO only pedigree, not using any age priors and constructing a full pedigree, and not using any age priors and constructing a PO only pedigree (Table S1).

Table S2. *Number of parent-offspring pairs identified by Sequoia with four distinct pedigrees.*

	Full pedigree<br>Age prior	Full pedigree<br>No age prior	PO only<br>Age prior	PO only<br>No age prior
Mother-offspring pairs (PO_M)	69	69	58	58
Father-offspring pairs (PO_P)	62	74	54	66

Most parent-offspring pairs (69%) were found in all four pedigrees (Figure S2). The vast majority of pairs found only in the models in which age prior was turned off were pairs in which the parent was too young to be sexually mature when the offspring was born (3 years old for females and 4 years old for males, to account for delayed implantation). The models that constructed a full pedigree identified more parent-offspring pairs. Using the full pedigree in Sequoia is more efficient and reliable for finding related pairs because it considers the likelihoods of all possible relationships jointly, rather than just evaluating each pair in isolation. Pairs found in only one of the model runs were more likely to have a log likelihood ratio (LLR) < 0.

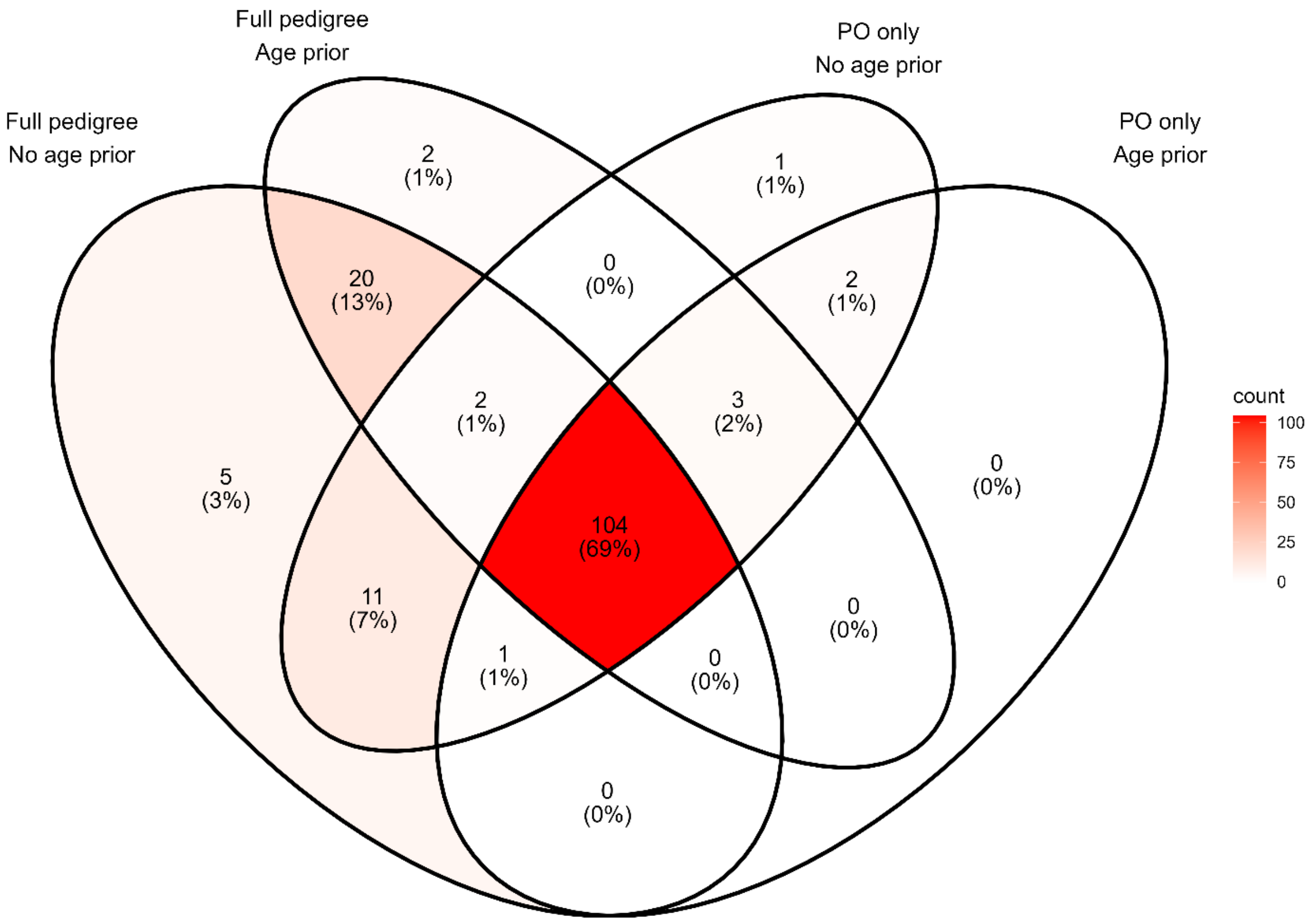

Figure S3. *Overlap of parent-offspring (PO) pairs identified across four pedigree reconstruction models.*

In close-kin mark–recapture (CKMR), related pairs must be identified from a large pool of mostly unrelated pairs, so statistical controls are needed to limit incorrect classifications (Skaug et al. 2010). Because false positives (and to a lesser extent false negatives) can affect CKMR estimates, minimizing both types of error is essential. Ultimately, we chose to use the pedigree where age priors were turned on and where we constructed the full pedigree, which we had the most confidence in. It is important to note that we excluded 4 mother-offspring pairs identified by Sequoia from the CKMR analysis because the potential mother was sampled in the same year the potential offspring was born (implying the possible harvest of a mother with her cub-of-the-year, which was prohibited in Michigan). The majority of pairs, 80%, had log likelihood ratio > 0, indicating high confidence in the identified pairs (Figure S3).

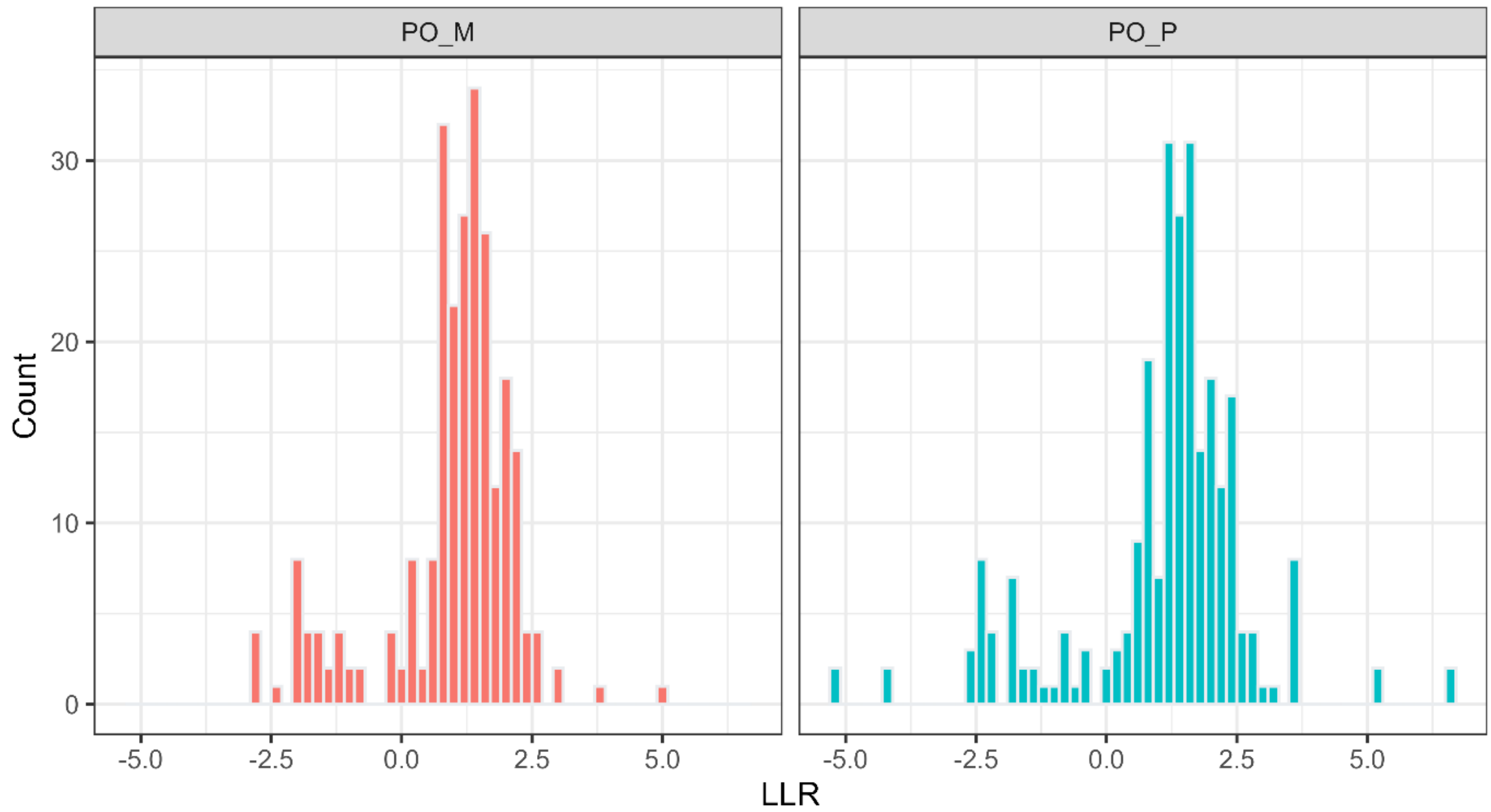

Figure S4. *Distribution of log likelihood ratios (LLR) for the mother-offspring (PO_M) and father-offspring (PO_P) pairs identified in the model with full pedigree and age priors. Sequoia attempts to maximize overall pedigree LLR, so having some pairs with LLR < 0 is acceptable.*

We found that pairs tended to be geographically close to each other, and 92% of pairs were found in either the same county or in counties that immediately bordered each other (Figure S4). We also determined that pairs were evenly distributed across bear management units.

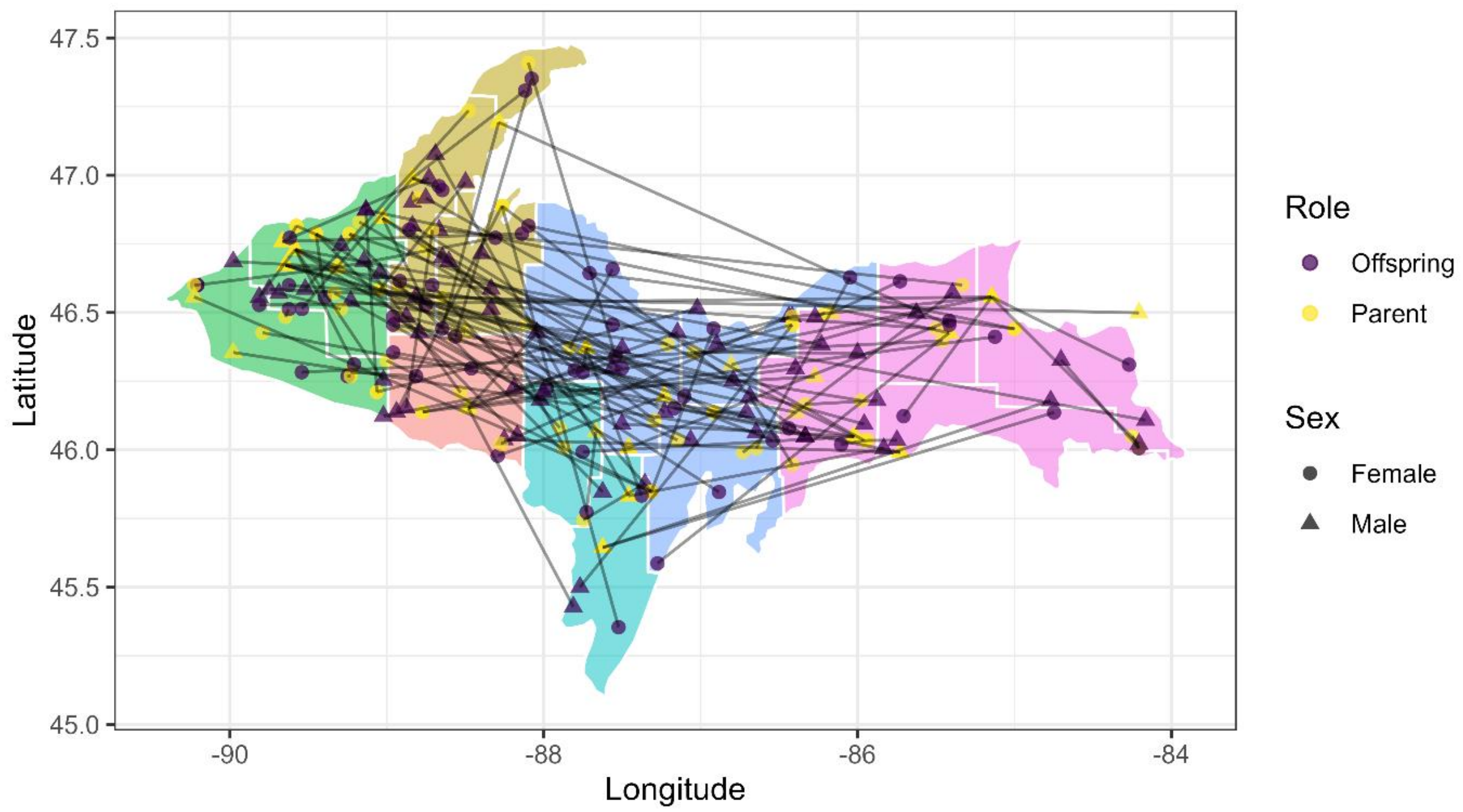

Figure S5. *Map of the parent-offspring pairs identified in the model with full pedigree and age priors. Counties are represented in different colors.*

In addition to the PO pairs, we found 121 full-sibling (FS), 17 maternal half-sibling (HS_M), and 102 paternal half-sibling (HS_P) pairs. Based on preliminary predictions from initial close-kin mark recapture (CKMR) simulations, these estimates seem less credible than the parent-offspring pair counts. CKMR simulations predicted significantly fewer FS pairs and significantly more HS pairs than the pedigree construction found. Additionally, our earlier power analysis demonstrated that we had low confidence in the ability to discern HS relationships, so it is unsurprising that the inferred sibling relationship counts are inaccurate. One possible explanation for why we observe so many more FS is that paternal half-siblings may have mothers who are closely related (i.e., siblings or mother-daughter), which could lead to misclassification.

References:

Anderson, E. C. 2024. CKMRsim: Inference of pairwise relationships using likelihood ratios. R package version 0.1.3.

Huisman, J. 2017. Pedigree reconstruction from SNP data: parentage assignment, sibship clustering and beyond. Molecular Ecology Resources 17:1009–1024.

Skaug, H. J., M. Bérubé, and P. J. Palsbøll. 2010. Detecting dyads of related individuals in large collections of DNA-profiles by controlling the false discovery rate. Molecular Ecology Resources 10:693–700.
