# Supplementary material for: Application of close‐kin mark–recapture to an American black bear population using harvest samples
Source: Ecol Appl. 2026 Oct 5;36(7):e70322. doi: 10.1002/eap.70322 (PMC13639227; doi:10.1002/eap.70322)
Supplement: Supplementary file 2 [file EAP-36-e70322-s002.pdf]

**Appendix S2: Age distribution of Michigan American black bears (*Ursus americanus*)**

# Application of close-kin mark–recapture to an American black bear population using harvest samples

Anthony Sévêque, Robert C. Lonsinger, Lisette P. Waits, Kristin E. Brzeski, Sarah L. Mayhew, D. Cody Norton, Caitlin N. Ott-Conn, Tyler R. Petroelje, Anaïs K. Tallon, Maya Weissman & Dana J. Morin

*Ecological Applications*

*Any use of trade, firm or product names is for descriptive purposes only and does not imply endorsement by the U.S. Government.*

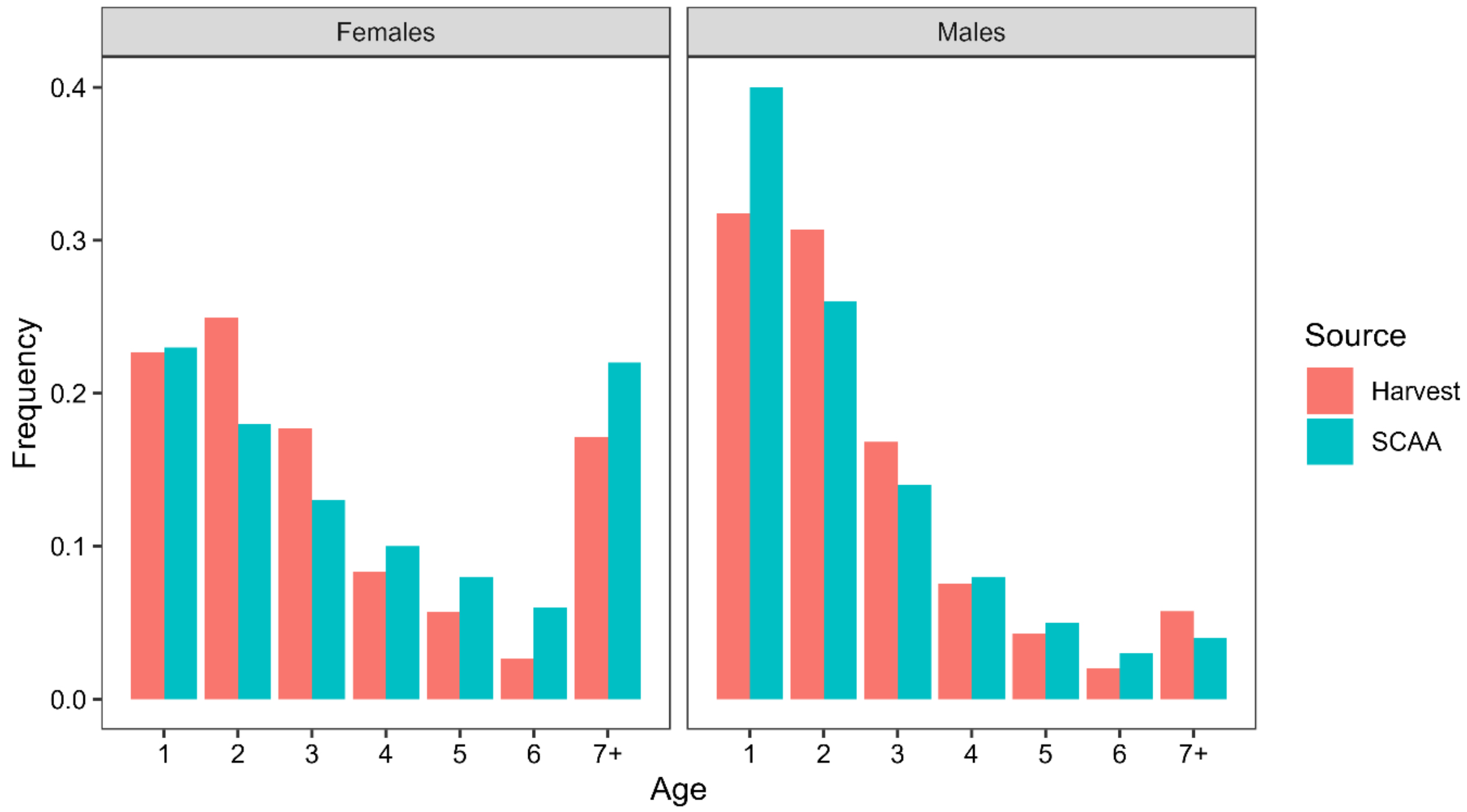


***Figure S1.*** *Age distribution of male and female black bears (Ursus americanus) in the Upper Peninsula of Michigan, USA, based on final samples from the 2021–2022 harvest season (red) or a previous statistical-catch-at-age model (SCAA; blue).*

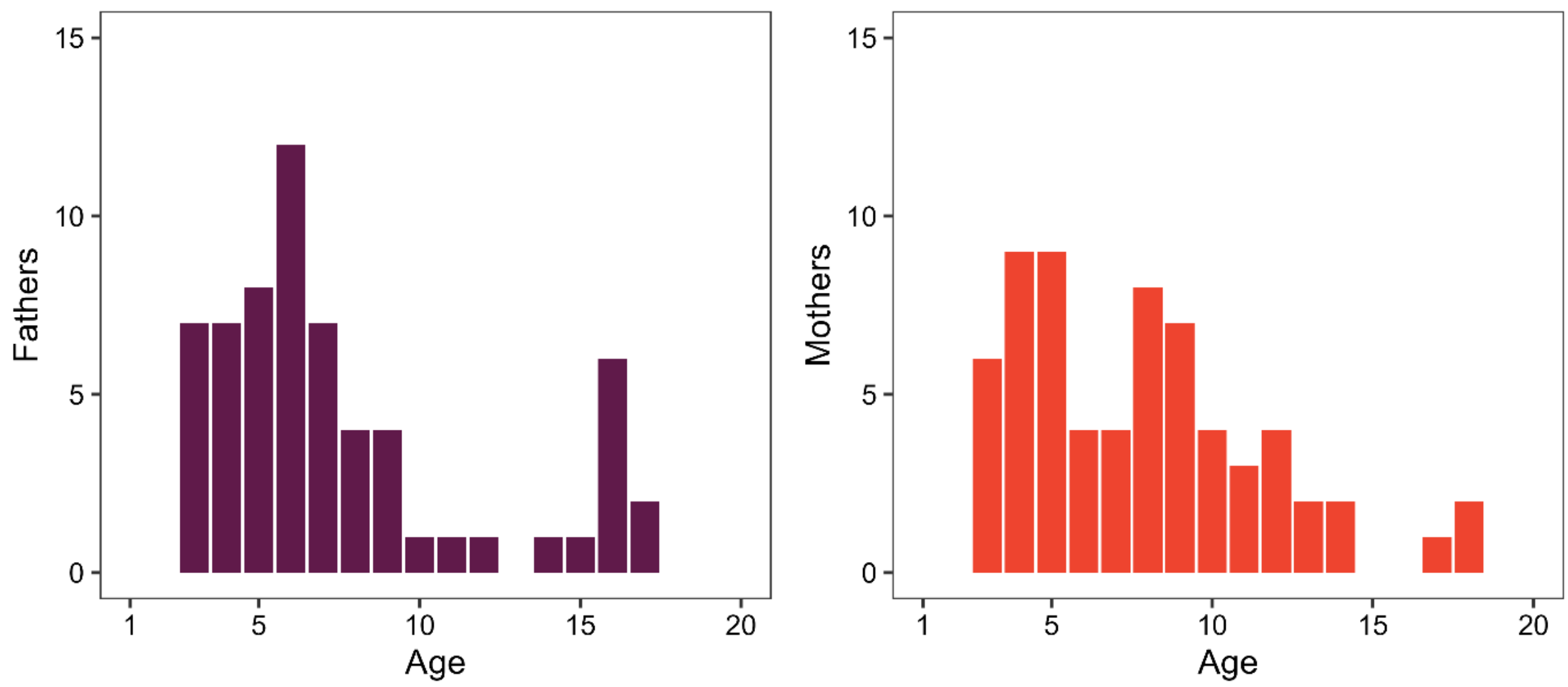


***Figure S2.*** *Age distribution of father and mother black bears (Ursus americanus) in the Upper Peninsula of Michigan, USA, from the identified set of parent-offspring pairs in samples collected from individuals harvested during 2021–2022.*
